# Integration of single-cell and bulk RNA sequencing reveals programmed cell death-associated transcriptional programs in sepsis-induced acute lung injury

**DOI:** 10.1371/journal.pone.0349288

**Published:** 2026-06-03

**Authors:** Xin Li, Weihong Liu, Yucui Zhang, Guohan Xiang, Qianyu Bi, Xin Lin, Yang Liu, Tejin Ba, Li Kong, Yang Liu, Hao Hao

**Affiliations:** 1 Shandong University of Traditional Chinese Medicine, Jinan, Shandong, China; 2 Affiliated Hospital of Shandong University of Traditional Chinese Medicine, Jinan, Shandong, China; 3 Inner Mongolia Autonomous Region International Mongolian Medical Hospital, Hohhot, China; Versiti Blood Research Institute, UNITED STATES OF AMERICA

## Abstract

**Background:**

Sepsis-induced acute lung injury (ALI) is a frequent and life-threatening complication of sepsis, yet clinically actionable transcriptomic biomarkers remain limited. Regulated/programmed cell death (PCD) pathways shape inflammatory injury and barrier dysfunction, but their cell-type-specific transcriptional signatures in sepsis-induced ALI are incompletely defined.

**Methods:**

Bulk transcriptomes from E-MTAB-5273 (training; sepsis-induced ALI vs sepsis) and E-MTAB-5274 (external validation) and scRNA-seq data from GSE207651 (CLP vs sham) were analyzed. Thirteen curated PCD gene sets were scored by ssGSEA, and differential PCD pathways were identified using the Wilcoxon rank-sum test with multiple-testing correction. WGCNA and differential expression analysis (limma) were integrated to obtain differentially expressed PCD-related genes (DE-PCDRGs). We benchmarked 113 machine-learning model combinations (12 algorithms) under cross-validation to select an optimal classifier, and interpreted predictions using SHAP and LIME. Associations between the PCD score and immune/metabolic signatures were assessed by ssGSEA. Cell types enriched for model-gene expression were localized in scRNA-seq, and key genes were validated in a CLP-induced ALI rat model using Western blot and immunohistochemistry.

**Results:**

Five PCD processes differed between sepsis-induced ALI and sepsis, including increased apoptosis and pyroptosis and decreased lysosome-dependent cell death, NETosis, and alkaliptosis. Twelve DE-PCDRGs were identified, and an 8-gene signature (PADI4, IFI6, POLB, IFI27, GZMB, CD3E, CRIP1, CASP5) yielded the best performance (glmBoost feature selection + random forest classifier; AUC 0.988 in training and 0.817 in validation). Enrichment analyses linked model genes to ribosome-related pathways, cell adhesion molecules, and the intestinal immune network for IgA production. High vs low PCD score groups differed in 11 immune-cell signatures and 23 metabolic pathways. Single-cell analyses highlighted endothelial cells as a major compartment expressing multiple model genes. In vivo experiments confirmed differential protein abundance of PADI4, POLB, and IFI27 in CLP-induced ALI lungs, supporting their potential as biomarkers.

**Conclusion:**

Integrating bulk and single-cell transcriptomes delineated PCD-associated molecular features in sepsis-induced ALI and identified an externally validated 8-gene classifier signature. These results nominate endothelial-cell-linked PCD programs and the PADI4/POLB/IFI27 axis for further mechanistic studies and biomarker development.

## 1. Introduction

Sepsis is a syndrome characterized by a dysregulated host response to invading pathogens, leading to multiple life-threatening organ dysfunction [1]. Among all complications of sepsis, acute lung injury (ALI) is one of the earliest and most severe [2]. The treatment of sepsis-induced ALI therefore remains a major challenge in reducing sepsis-related morbidity and mortality [3]. Despite progress in understanding the pathophysiology of sepsis, there remains a lack of robust molecular tools for the early identification and risk stratification of sepsis-induced ALI [4,5]. Developing transcriptome-based classifiers may therefore improve both mechanistic understanding and clinical risk assessment.

Programmed cell death (PCD) is a genetically regulated process that eliminates damaged or exhausted cells and plays a crucial role in both homeostasis and disease [6–8]. Excessive or dysregulated cell death can amplify inflammatory injury, tissue destruction, and organ dysfunction [9]. In ALI, multiple PCD pathways may damage both alveolar epithelial and endothelial cells and exacerbate barrier dysfunction [10]. Beyond apoptosis, several regulated cell-death modalities – including pyroptosis, necroptosis, ferroptosis, and NETosis - have been implicated in sepsis and lung injury through cytokine release, immunothrombosis, and disruption of the alveolar epithelial-endothelial barrier. These pathways are increasingly viewed as potential therapeutic nodes, underscoring the value of defining PCD-associated gene programs and the cell types in which they operate during sepsis-induced ALI.

Bulk RNA-seq captures disease-associated transcriptional changes but cannot resolve cellular heterogeneity, whereas scRNA-seq attributes signals to specific cellular compartments. Integrating these modalities can therefore prioritize robust biomarkers and nominate candidate effector cell types for subsequent functional validation. Machine learning (ML) further enables high-dimensional feature selection and model building from transcriptomic data [11,12].

Here, we integrated bulk RNA-seq and scRNA-seq to (i) quantify PCD pathway activity in sepsis-induced ALI, (ii) identify differentially expressed PCD-related genes through network and differential-expression analyses, (iii) develop and externally validate a transcriptomic classification model, and (iv) localize key model genes at single-cell resolution. Finally, we validated the expression of selected model genes (PADI4, POLB and IFI27) in a CLP-induced ALI animal model to support the robustness of the identified signature, rather than to infer a complete molecular mechanism.

## 2. Materials and methods

### 2.1. Data source

Two human mRNA expression matrices for sepsis-induced ALI were obtained from ArrayExpress. E-MTAB-5273 served as the training set and comprised 127 sepsis-induced ALI samples and 94 sepsis samples. E-MTAB-5274 served as the external validation set and contained 54 sepsis-induced ALI samples and 54 sepsis samples. In addition, scRNA-seq data were retrieved from GEO under accession GSE207651, which included two cecal ligation and puncture (CLP) mouse samples and one sham-control sample.

The datasets were selected according to the following criteria: (i) relevance to sepsis-induced ALI, (ii) availability of high-quality transcriptomic data with relatively adequate sample size, (iii) clear grouping information for disease and control conditions, and (iv) acceptable platform comparability to reduce technical bias. We acknowledge that these datasets do not fully represent the entire heterogeneity of all sepsis-induced ALI patients. Accordingly, the present study should be viewed as a discovery-and-validation framework built on the best currently available public datasets rather than as a definitive representation of all ALI populations. Because sepsis-induced ALI is a systemic inflammatory syndrome, peripheral-blood transcriptomes remain clinically relevant for biomarker discovery and risk stratification; tissue-level context was then added using lung scRNA-seq and animal validation.

The 13 PCD categories and gene counts were as follows: alkaliptosis (7), apoptosis (580), autophagy (367), cuproptosis (14), disulfidptosis (4), entotic cell death (15), ferroptosis (88), lysosome-dependent cell death (220), necroptosis (101), NETosis (8), oxeiptosis (5), parthanatos (9), and pyroptosis (52). In addition, 83 metabolic pathways encompassing 1,685 metabolism-related genes were downloaded from KEGG [13]. Processed expression matrices and corresponding phenotype annotations were downloaded. Gene identifiers were mapped to official gene symbols; when multiple probes mapped to the same gene, the median expression value was used. Expression values were log2-transformed when necessary.

### 2.2. Identification of PCD pathways associated with sepsis-induced ALI

Enrichment scores for each of the 13 PCD types in each sample were calculated using the ssGSEA algorithm from the R package GSVA, based on gene-expression profiles from all samples in the training set. Differences between sepsis-induced ALI and sepsis samples were visualized using violin plots and tested using the Wilcoxon rank-sum test with Benjamini-Hochberg FDR correction. Random forest analysis (ntree = 500, default mtry) was then used to rank the relative contribution of differential PCD types. The 13 PCD gene sets were curated from published literature.

### 2.3. Identification of PCD-related module genes through weighted gene co-expression network analysis (WGCNA)

All samples in the training set were subjected to hierarchical clustering using the hclust function to identify and exclude abnormal samples. Network topology analysis was performed using pickSoftThreshold, with the criterion of R2 > 0.85 to determine the soft-thresholding power for approximate scale-free topology. A signed co-expression network was constructed using the WGCNA package. Modules were detected with dynamic tree cutting (minModuleSize = 30) and merged at mergeCutHeight = 0.25.

Associations between module eigengenes and differential PCD pathways were assessed using Pearson correlation analysis. According to the correlation coefficient and p value, the most positively or negatively associated modules for each differential PCD type were selected as key modules. Genes with gene significance (GS) > 0.2 and module membership (MM) > 0.4 were retained as module genes. These thresholds were used at the discovery stage to balance stringency and recall; final candidates were further restricted by DEG intersection and machine-learning feature selection.

### 2.4. Construction and validation of classification models

Differentially expressed genes (DEGs) between sepsis-induced ALI and sepsis in the training set were identified using limma, with |log2FC| > 0.5 and FDR < 0.05 as significance thresholds. DE-PCDRGs were then obtained by intersecting PCD-related module genes, genes associated with differential PCD pathways, and DEGs.

To develop a robust classifier, 113 combinations of algorithms were tested using 12 machine-learning methods: LASSO, Ridge, Elastic Net, Stepglm, SVM, glmBoost, LDA, plsRglm, RF, GBM, XGBoost, and Naive Bayes. In each combination, one algorithm was used for variable selection and another for model construction. Model development in the training set used repeated k-fold cross-validation (10 folds, 5 repeats) with a fixed random seed, and the independent dataset served as an external validation set. AUC, calibration, and decision-curve analysis were used to evaluate performance.

### 2.5. Construction of a nomogram for risk estimation

The model genes selected by the optimal machine-learning pipeline were used to construct a nomogram with the rms package to estimate the probability of progression from sepsis to sepsis-induced ALI. ROC analysis, calibration curves, and decision-curve analysis were used to assess its predictive value. Calibration was assessed by bootstrap resampling (1,000 resamples), and the optimal probability threshold was determined by the Youden index in the training set and then applied to the validation set.

### 2.6. Correlation analysis and gene set enrichment analysis

Spearman correlation among model genes was calculated. Gene Set Enrichment Analysis (GSEA) was then performed for the model genes using clusterProfiler with 1,000 permutations. Pathways with FDR < 0.25 and nominal P < 0.05 were considered enriched. The background gene set was c2.cp.kegg.v7.5.1.symbols.gmt from MSigDB.

### 2.7. Effect of the PCD score on the immune and metabolic microenvironment of sepsis-induced ALI

A PCD score for each sample in the training set was calculated using ssGSEA on the 8 model genes. Samples were dichotomized into high-score and low-score groups according to the median value. Enrichment scores for 28 immune-cell signatures and 83 metabolic pathways were also calculated using ssGSEA. The Wilcoxon test with FDR correction was used to compare immune-cell signatures, metabolic pathways, and immune activation/suppression genes between the two groups.

### 2.8. Construction of TF-mRNA-drug network and molecular docking analysis

Transcription factors related to the model genes were obtained from miRNet, and differentially expressed TFs were identified through expression analysis. Drugs with an interaction score > 0.5 with the model genes were selected from DGIdb. The TF-mRNA-drug network was visualized in Cytoscape. Candidate TF-gene interactions were filtered by evidence level and intersected with differentially expressed TFs.

Protein structures were downloaded from UniProt, and three-dimensional drug structures were obtained from PubChem. Molecular docking was performed using CB-Dock, based on the AutoDock Vina algorithm. Proteins were preprocessed by removing water molecules and adding hydrogen atoms. Docking results were treated as hypothesis-generating and require experimental confirmation.

### 2.9. Processing of scRNA-seq data

Quality control was first performed on GSE207651. To enable cross-species integration between the human bulk cohorts and the rodent scRNA-seq/animal validation, gene symbols were harmonized by mapping rodent genes to human one-to-one orthologs using Ensembl BioMart. Genes without unambiguous one-to-one orthologs were excluded from signature scoring to avoid erroneous cross-species mapping; consequently, species-specific regulators may have been missed.

Cells were retained with 100 < nFeature < 2500, nCount < 10000, and mitochondrial gene proportion < 5%. Data were normalized using SCTransform, followed by PCA and UMAP for dimensionality reduction and clustering (resolution = 0.5). Marker genes were identified with the Wilcoxon test with FDR correction. Cell types were annotated according to canonical markers, SingleR reference mapping, and the CellMarker 2.0 database [14]. CellChat was used to analyze receptor-ligand interactions among cell clusters, and Monocle2 was used for pseudotime analysis.

### 2.10. Animal experiments

All animal experiments involving sepsis models in rats were conducted and reported in accordance with ARRIVE 2.0 guidelines. Experiments were approved by the Experimental Animal Welfare and Ethics Committee of Shandong University of Traditional Chinese Medicine (approval number: SDSZYYAWE20231220001). SPF male Sprague-Dawley rats (6−8 weeks old, 200−300 g) were purchased from Beijing Vital River Laboratory Animal Technology Co., Ltd. and housed under standard conditions (22 + /- 2 degrees C, 50 + /- 5% humidity, 12-h light/dark cycle).

Rats were randomly divided into a sham group and a CLP group (n = 6 each). The CLP-induced sepsis/ALI model was established under isoflurane anesthesia. The cecum was ligated at the midpoint and punctured three times using a 16-gauge needle. Sham-operated rats underwent laparotomy and cecal mobilization without ligation or puncture. Saline was administered subcutaneously after surgery. Humane euthanasia was performed via carbon dioxide inhalation at the end of the study.

### 2.11. Histological analysis

Lung tissues were fixed in 4% paraformaldehyde, embedded in paraffin, sectioned at 4 um, and stained with hematoxylin and eosin. Lung injury scoring included edema, hemorrhage, neutrophil infiltration, and hyaline membrane formation (0–4 for each component). Scores were evaluated by two blinded technicians.

### 2.12. Western blotting

Proteins were isolated from lung tissues, separated by SDS-PAGE, and transferred to PVDF membranes. The membranes were then probed with the following primary antibodies: anti-PADI4 (diluted 1:2000; ORIGENE, TA504813), anti-POLB (diluted 1:3000; ABclonal, A2412), anti-IFI27 (diluted 1:1000; GeneTex, GTX66233), and anti-GAPDH (diluted 1:10000; Proteintech, 60004–1-IG). Antibodies were incubated at 4 °C overnight. Then incubation with horseradish peroxidase-conjugated secondary antibodies (diluted 1: 5000；ABclonal，AS014）. Bands were detected using an ECL kit (Beyotime, P0018S) and analyzed with ImageJ software. GAPDH was used as an internal control.

### 2.13. Immunohistochemical staining

Paraffin-embedded lung tissue sections were incubated at 37 °C overnight. Subsequently, the sections were incubated in 3% hydrogen peroxide at room temperature in the dark. The sections were heated under microwave treatment and allowed to cool naturally for 40 minutes. After antigen retrieval, samples were blocked with 1% BSA for 30 min, followed by overnight incubation at 4 °C with anti-PADI4 (diluted 1:1800; ORIGENE, TA504813) and anti-POLB (diluted 1:3000; ABclonal, A2412). After washing, anti-rabbit HRP secondary antibody (1:10000; Abcam, ab205718) was incubated at room temperature for 1 hour, followed by incubation with an avidin-biotin-peroxidase complex for 15 minutes and diaminobenzidine staining for 5 minutes. Specimens were analyzed under a light microscope.

### 2.14. Statistical analysis

Statistical analyses for bulk RNA-seq, scRNA-seq, and machine learning were performed in R (version 4.x). For ssGSEA-based pathway scores and immune/metabolic signatures, between-group comparisons used the Wilcoxon rank-sum test with Benjamini-Hochberg correction. Differential expression was analyzed using limma, with |log2FC| > 0.5 and FDR < 0.05 considered significant. Module-trait correlations in WGCNA were assessed by Pearson correlation. Model performance was evaluated by AUC using pROC, with 95% confidence intervals calculated by DeLong’s method. Animal experiments were analyzed in GraphPad Prism; data are shown as mean + /- SEM and compared using an unpaired two-tailed t test or Mann-Whitney U test where appropriate. A two-sided P < 0.05 was considered significant.

## 3. Results

### 3.1. Identification of PCD-related module genes in sepsis-induced ALI

**Fig 1:**
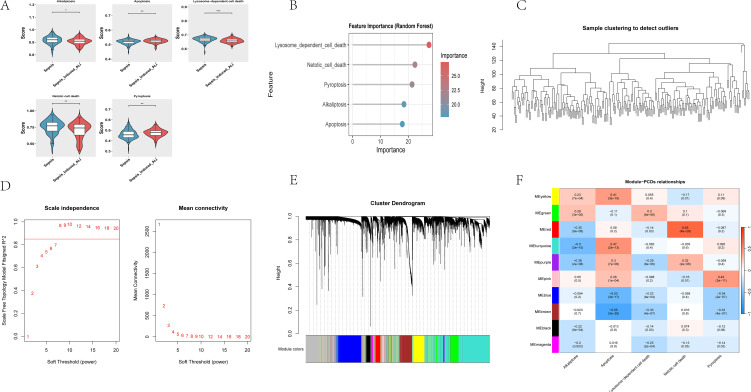
Screening of differential PCD types and related module genes. (A) ssGSEA enrichment scores of the five differential PCD pathways in the training set (E-MTAB-5273). **(B)** Random forest-based importance ranking of the five differential PCD pathways. **(C)** Sample clustering dendrogram for WGCNA quality control; each branch represents one sample and the y-axis indicates Euclidean distance. **(D)** Soft-thresholding power selection for WGCNA. **(E)** Module dendrogram and module color assignment. **(F)** Module-trait correlation heatmap between module eigengenes and differential PCD pathway scores‌‌.

In the training set, five PCD pathways differed significantly between sepsis-induced ALI and sepsis (Fig 1A). Compared with sepsis samples, apoptosis and pyroptosis scores were increased, whereas alkaliptosis, lysosome-dependent cell death, and NETosis scores were decreased. Random forest ranking suggested that lysosome-dependent cell death and NETosis contributed most strongly to the separation between groups (Fig 1B).

Because ssGSEA yields relative enrichment scores rather than direct measurements of pathway flux, the lower NETosis score should be interpreted cautiously. In this blood-based cohort, it most likely indicates that NETosis-related transcripts were comparatively less dominant than other inflammatory programs at the sampled time point, rather than proving that NET formation is universally reduced in lung tissue.

WGCNA sample clustering identified no outlier samples. Fig 1C shows the sample dendrogram used for quality control, in which each branch represents one sample and the vertical axis represents Euclidean distance. The soft-thresholding analysis indicated that beta = 8 achieved a scale-free topology fit index (R2) > 0.85, and 10 co-expression modules were identified (Fig 1C-E). In Fig 1F, module eigengenes (MEs) summarize the expression pattern of each co-expression module; thus, terms such as MEturquoise refer to module eigengenes rather than to prediction models. The heatmap indicates which modules are most strongly associated with each differential PCD pathway and therefore guides the selection of biologically relevant modules for downstream screening. Specifically, apoptosis showed a positive correlation with the turquoise module in Fig 1F, apoptosis and lysosome-dependent cell death were most strongly associated with the brown module, NETosis with the red module, and pyroptosis with the pink module. These four modules were therefore retained as key PCD-related modules, yielding 5,460 module genes after applying GS > 0.2 and MM > 0.4.

### 3.2. Identification of DE-PCDRGs and establishment of a sepsis-induced ALI classifier

**Fig 2:**
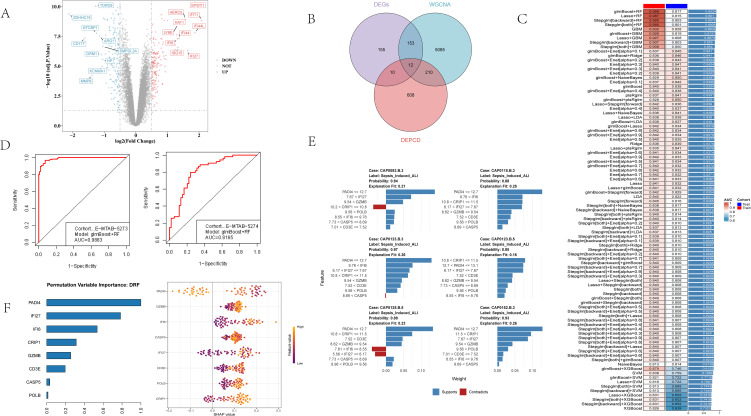
Screening of DE-PCDRGs and construction of the optimal model. **(A)** Volcano plot of DEGs between sepsis-induced ALI and sepsis in the training set. **(B)** Venn diagram showing the intersection yielding 12 DE-PCDRGs. **(C)** Heatmap of AUC values for 113 model combinations. **(D)** ROC curves of the optimal model in the training and validation sets. **(E)** Representative LIME explanations for the RF classifier. **(F)** SHAP summary plot showing the contribution and directionality of each model gene to predicted risk.

In the training set, 330 DEGs were identified, including 169 upregulated and 161 downregulated genes (Fig 2A). Intersecting the 5,460 PCD-related module genes, 840 genes corresponding to differential PCD pathways, and 330 DEGs yielded 12 DE-PCDRGs (Fig 2B). We then evaluated 113 algorithm combinations built from 12 machine-learning methods. In each combination, one algorithm was used for feature selection and another for model construction. The cross-validated AUC heatmap (Fig 2C) showed that the glmBoost + RF combination performed best.

In this framework, glmBoost served as the feature-selection step and reduced the 12 DE-PCDRGs to 8 genes (PADI4, IFI6, POLB, IFI27, GZMB, CD3E, CRIP1, and CASP5), after which RF was used as the final classifier. Because this combination showed the best overall and most stable performance across resampling and external validation, subsequent interpretation focused on the final RF model rather than on all tested algorithms.

Using these 8 genes, the RF classifier achieved an AUC of 0.988 in the training set and 0.817 in the external validation set (Fig 2D). The reduction in AUC from training to validation suggests some degree of model optimism, which is common in high-dimensional transcriptomic analyses; however, the validation performance remained acceptable and did not collapse outside the training data. Six representative LIME explanations showed that the predicted probability of sepsis-induced ALI exceeded 85% in the displayed high-risk examples (Fig 2E). SHAP analysis ranked the genes as follows: PADI4, IFI27, IFI6, CRIP1, GZMB, CD3E, CASP5, and POLB. Higher PADI4 expression tended to shift predictions toward lower risk, whereas higher expression of the remaining seven genes shifted predictions toward higher risk (Fig 2F). Overall, these findings suggest a bidirectional signature in which PADI4 behaves as a putative protective marker, while the remaining genes act as risk-associated features.

### 3.3 Visualization of the RF classifier

**Fig 3:**
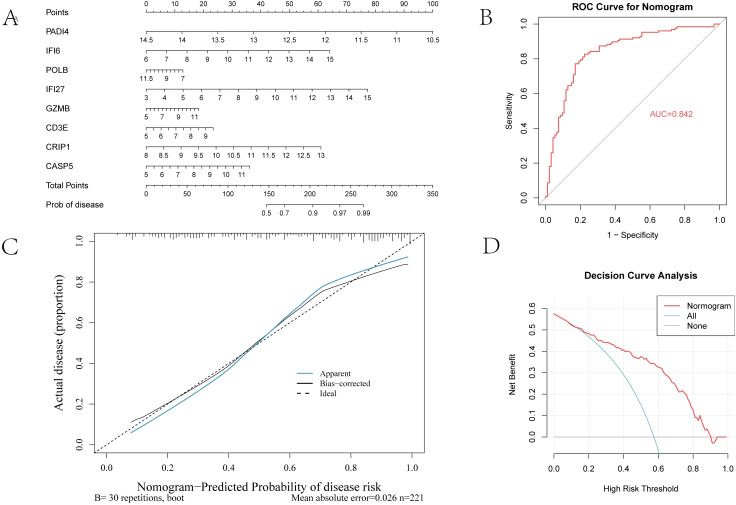
Nomogram and evaluation of the RF model. **(A)** Nomogram constructed from the 8-gene classifier signature. **(B)** ROC curve of the nomogram in the validation set. **(C)** Calibration curve. **(D)** Decision-curve analysis.

The nomogram was constructed from the same 8 genes retained in the optimal glmBoost + RF pipeline, because these genes represented the most stable and informative feature set after sequential filtering by WGCNA, differential expression, and machine learning. In the nomogram, each gene has its own point scale because the scales reflect the relative contribution (coefficient/weight) of that variable to the prediction model rather than a common unit of measurement. For each patient, the expression value of a given gene is projected to its corresponding points, the points from all 8 genes are summed to yield total points, and the total points are then converted into the predicted probability of sepsis-induced ALI (Fig 3A). Thus, the individual gene scales should not be compared directly; instead, the nomogram should be interpreted as a cumulative scoring system.

The nomogram achieved an AUC of 0.842 (Fig 3B), showed good agreement between predicted and observed risk in the calibration plot (Fig 3C), and demonstrated net clinical benefit in decision-curve analysis (Fig 3D).

### 3.4. Characterization of model genes in sepsis-induced ALI

**Fig 4:**
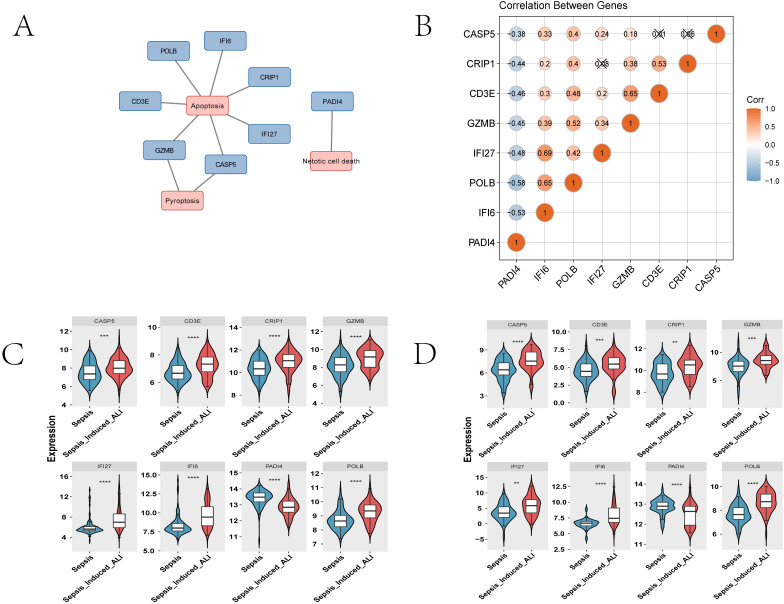
Characteristic analysis of model genes. **(A)** Distribution of PCD pathway annotations for the 8 model genes. **(B)** Spearman correlation matrix of model-gene expression. **(C-D)** Boxplots comparing expression of model genes between groups in the training and validation sets.

We next summarized the PCD annotations represented by the 8 model genes. Six genes were annotated to apoptosis-related gene sets, among which GZMB and CASP5 were additionally linked to pyroptosis, whereas PADI4 was annotated to NETosis (Fig 4A). Thus, the final signature spans more than one PCD modality rather than representing a single-death-pathway model.

Spearman correlation analysis showed that PADI4 was significantly negatively correlated with each of the other 7 model genes (cor < −0.3, p < 0.05), with the strongest negative correlation observed between POLB and PADI4 (cor = −0.58). The remaining genes showed varying degrees of positive correlation, with the strongest positive correlation between IFI27 and IFI6 (cor = 0.69) (Fig 4B). Wilcoxon analysis further showed consistent expression trends in the training and validation cohorts: PADI4 was significantly downregulated in sepsis-induced ALI, whereas the other 7 genes were significantly upregulated (Fig 4C-D). In addition, 4 genes (PADI4, IFI6, POLB, and IFI27) each achieved an AUC > 0.7 in both cohorts (S1 Fig).

### 3.5. GSEA revealed potential mechanisms of model genes involved in sepsis-induced ALI

Through GSEA, CRIP1, GZMB, PADI4, POLB, and CD3E were enriched in ribosome-related pathways. PADI4, POLB, and CRIP1 were additionally associated with cell adhesion molecules, whereas CRIP1 and PADI4 were linked to the intestinal immune network for IgA production. IFI6 and POLB were jointly enriched in antigen processing and presentation. IFI27 was associated with regulation of the actin cytoskeleton, whereas CASP5 was enriched in the toll-like receptor signaling pathway and chemokine signaling pathway (S2 Fig).

### 3.6. Association of the 8-gene PCD signature with the immune and metabolic microenvironment in sepsis-induced ALI

**Fig 5:**
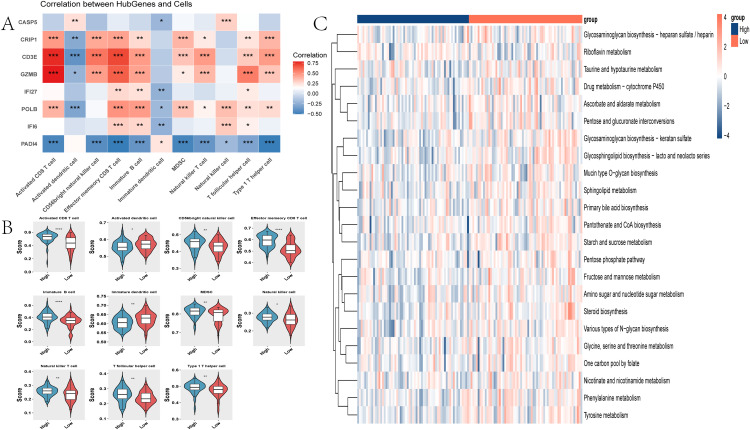
Association of the 8-gene PCD signature with the immune and metabolic microenvironment in sepsis-induced ALI. **(A)** Correlation heatmap between the eight model genes and the 11 immune-cell signatures that differed between the high and low PCD score groups. **(B)** Differences in ssGSEA-inferred immune-cell signature scores between the high and low PCD score groups. **(C)** Differences in ssGSEA-inferred metabolic pathway scores between the high and low PCD score groups‌‌.

To further clarify how the 8-gene signature relates to the immune landscape of sepsis-induced ALI, we first analyzed the correlations between the eight model genes and the 11 immune-cell signatures that differed between the high and low PCD score groups. As shown in Fig 5A, several model genes exhibited significant associations with multiple immune-cell signatures, indicating that the classifier signature is closely linked to immune remodeling in sepsis-induced ALI. In particular, genes associated with increased disease risk tended to correlate with activated immune-cell populations, whereas PADI4 showed an opposite trend, consistent with its distinct contribution pattern in the model.

Next, samples in the training cohort were divided into high and low PCD score groups according to the median PCD score, and differences in immune-cell infiltration were evaluated. We observed significant differences in 11 immune-cell signatures between the two groups, including activated CD8 T cells, activated dendritic cells, CD56bright natural killer cells, effector memory CD8 T cells, immature B cells, immature dendritic cells, myeloid-derived suppressor cells (MDSCs), natural killer T cells, natural killer cells, T follicular helper cells, and type 1 T helper cells (Fig 5B). These findings suggest that a higher PCD score is accompanied by broad alterations in both innate and adaptive immune compartments.

We further compared the expression of immune-regulatory genes between the two PCD score groups. Several immune-activation genes, including CD27, CD28, CD40, CD40LG, CXCL12, ICOS, and LTA, differed significantly between groups. Similarly, multiple immune-suppressive genes, including CD160, CD96, CSF1R, IDO1, IL10RB, LAG3, LGALS9, and TIGIT, also showed significant differences (S3 Fig). Together, these results indicate that the PCD signature is associated not only with changes in immune-cell composition but also with a broader shift in immune-regulatory status.

Because immune dysregulation in sepsis-induced ALI is often accompanied by metabolic reprogramming, we next assessed metabolic pathway activity in the two PCD score groups. Significant differences were observed in 23 metabolic pathways (Fig 5C), involving amino acid metabolism (glycine, serine and threonine metabolism, phenylalanine metabolism, and tyrosine metabolism), carbohydrate metabolism (fructose and mannose metabolism, starch and sucrose metabolism, pentose and glucuronate interconversions, and pentose phosphate pathway), vitamin metabolism (ascorbate and aldarate metabolism, riboflavin metabolism, nicotinate and nicotinamide metabolism, and pantothenate and CoA biosynthesis), and lipid-related pathways (glycosaminoglycan biosynthesis–heparan sulfate/heparin, keratan sulfate, glycosphingolipid biosynthesis–lacto and neolacto series, mucin type O-glycan biosynthesis, and sphingolipid metabolism). These results suggest that the 8-gene PCD signature reflects coordinated immunometabolic alterations in sepsis-induced ALI.

### 3.7. Prediction analysis of TFs, drugs, and model genes in sepsis-induced ALI

A total of 33 TFs and 35 drugs were predicted from the 8 model genes, and 11 TFs differed between the sepsis and sepsis-induced ALI groups. In the TF-mRNA-drug network, FOXC1 simultaneously regulated GZMB, CASP5, and CRIP1, whereas GATA2 simultaneously regulated GZMB, CD3E, and CRIP1. Thirteen drugs were predicted to target CD3E, including enavogliflozin, tofogliflozin anhydrous, teplizumab, and otelixizumab. Drugs targeting CRIP1 were mainly diacylglycerol lipase inhibitors such as DO34, KT-109, and DH376. No corresponding drugs were retrieved for IFI6 (S4 Fig).

Molecular docking results included CASP5-EMRICASAN (−7.8 kcal/mol), CD3E-SERGLIFLOZIN (−8.2 kcal/mol), CRIP1-DH376 (−6.6 kcal/mol), GZMB-GKT136901 (−8.4 kcal/mol), IFI27-VITAMIN D (−6.7 kcal/mol), PADI4-URIC ACID (−7.0 kcal/mol), and POLB-EDGEWORIN (−8.6 kcal/mol), indicating potentially favorable binding activity for the top-scoring drug-gene pairs (S5 Fig).

### 3.8. Alterations in sepsis-induced ALI at the single-cell level

**Fig 6:**
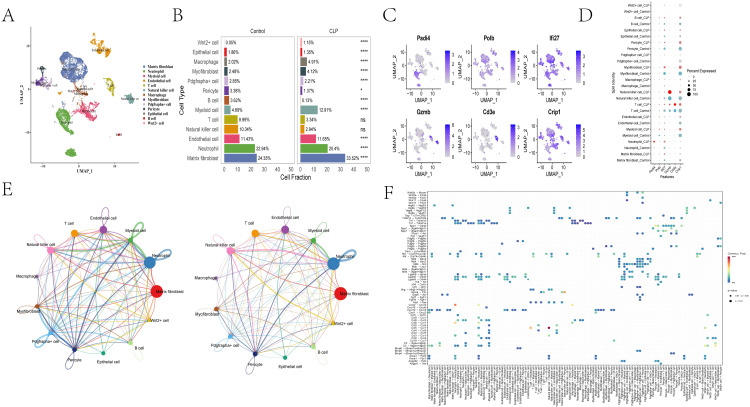
Characteristics at the single-cell level. **(A)** UMAP embedding of lung single cells with annotation of 13 major cell types. **(B)** Cell-type proportions by condition. **(C)** Violin/feature plots showing expression of model genes across cell types. **(D)** Cell communication network. **(E)** Predicted key ligand-receptor interactions involving endothelial cells.

A total of 15,638 high-quality cells were clustered into 22 clusters, and 13 major cell types were ultimately identified after annotation using canonical markers, SingleR, and the CellMarker 2.0 database [14], including matrix fibroblasts, neutrophils, myeloid cells, endothelial cells, T cells, natural killer cells, macrophages, myofibroblasts, Pdgfra+ cells, pericytes, epithelial cells, B cells, and Wnt2 + cells (Fig 6A and S6). Differential cell-type distributions between the control and CLP groups are shown in Fig 6B.

Because the single-cell data were derived from mouse samples, homology conversion was performed on the model genes, and six model genes (PADI4, POLB, IFI27, Gzmb, Cd3e, and Crip1) were successfully matched for subsequent analysis. Prominent expression of Crip1, IFI27, Gzmb, and POLB was observed in endothelial cells (Fig 6C and S7), suggesting that endothelial cells are an important compartment for the observed PCD-related signature in the lung scRNA-seq data. This observation does not exclude a pathogenic role for alveolar epithelial injury; rather, it indicates that endothelial PCD programs may be particularly relevant to barrier dysfunction and intercellular signaling in this dataset.

Cell communication analysis showed that endothelial cells had extensive crosstalk with other cell types, especially natural killer cells, matrix fibroblasts, and pericytes (Fig 6D). Ligand-receptor analysis suggested that endothelial cells communicated with these compartments mainly via Lgals9-Cd45, Pdgfb-Pdgfra, and Pdgfb-Pdgfrb, respectively (Fig 6E). Endothelial cells could be further divided into 6 subpopulations that were enriched in pathways such as epithelial cell migration, ameboid-type cell migration, collagen-containing extracellular matrix, IL-17 signaling, complement and coagulation cascades, and axon guidance. Pseudotime analysis also suggested clear differences in endothelial-state trajectories between the two groups (S8 Fig).

### 3.9. Validation of sepsis-induced ALI biomarkers

**Fig 7:**
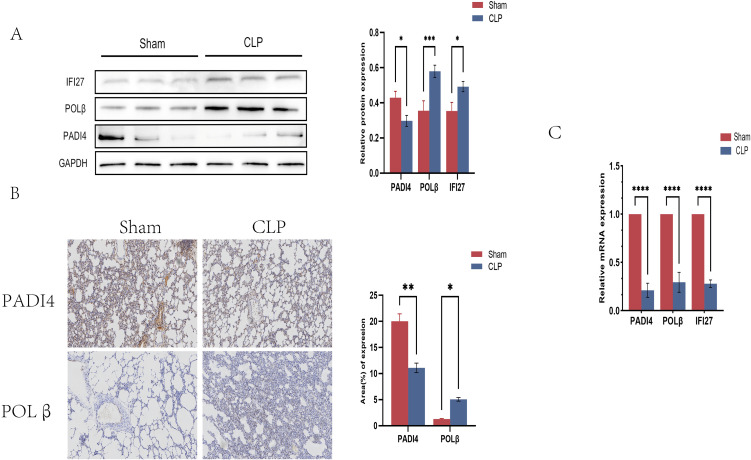
Verification of key gene expression by animal experiments. **(A)** Representative Western blots and densitometric quantification of PADI4, POLB, and IFI27 in lungs from CLP and sham rats. **(B)** Representative immunohistochemical staining for POLB and PADI4.

Based on gene-importance ranking, external validation performance, homologous matching in scRNA-seq, and reagent feasibility, PADI4, POLB, and IFI27 were selected for exploratory experimental validation as representative genes from the 8-gene signature. Sham-operated and CLP rats were established (n = 6 per group), and lung tissues were analyzed by Western blotting and immunohistochemistry. Western blotting showed that IFI27 and POLB protein abundance was increased in the CLP group, whereas PADI4 was decreased (P < 0.05; Fig 7A). Immunohistochemistry showed enhanced POLB staining and reduced PADI4 staining in the CLP group (Fig 7B). These findings support that representative signature genes are dysregulated in an experimental sepsis-induced ALI model.

**Fig 8:**
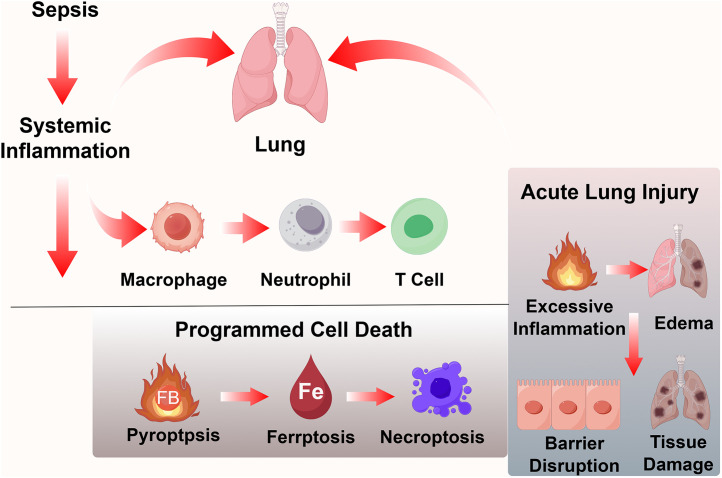
Proposed immunological mechanism linking programmed cell death programs to sepsis-induced acute lung injury.

Collectively, our findings suggest that sepsis triggers immune-cell activation and aberrant programmed cell death programs, which amplify inflammatory signaling and contribute to endothelial barrier disruption and lung injury. The proposed immunological model is summarized in Fig 8.

## 4. Discussion

This study integrated bulk transcriptomics, single-cell profiling, and in vivo validation to define PCD-associated molecular features in sepsis-induced ALI and to translate these signals into an externally validated classifier signature. Compared with prior studies that focused on only one or two cell-death modalities, our analysis jointly evaluated multiple regulated cell-death pathways, linked pathway activity to the immunometabolic microenvironment, and localized candidate biomarkers to specific lung cell compartments. These features improve interpretability while also generating hypotheses for downstream mechanistic studies.

Sepsis-induced ALI is driven by systemic inflammation, endothelial and epithelial barrier injury, and extensive immune dysregulation [15–17]. Against this background, we identified five PCD pathways with differential ssGSEA scores between sepsis-induced ALI and sepsis. Among them, the reduced NETosis score requires cautious interpretation. NETosis is often reported to increase in lung injury, but our score was derived from a peripheral-blood transcriptomic cohort and reflects relative enrichment of a small curated NETosis gene set rather than direct quantification of NET release in lung tissue. We therefore interpret the lower score as indicating that, at the sampled stage and in this blood-based dataset, NETosis-related transcripts were less dominant than other inflammatory programs such as apoptosis and pyroptosis, rather than as evidence against a pathogenic role for NETs in sepsis-induced ALI [18–21].

The selection of the human blood datasets also warrants clarification. Although ALI manifests in the lung, sepsis-induced ALI develops in the context of systemic host-response dysregulation, and blood transcriptomes provide a clinically accessible readout of immune activation and risk stratification. For that reason, we used E-MTAB-5273 as the discovery cohort and E-MTAB-5274 as the external validation cohort for biomarker identification, while lung scRNA-seq and animal experiments were incorporated to add tissue-level and mechanistic context. Even so, these cohorts do not capture the full heterogeneity of all sepsis-induced ALI patients, and our conclusions should therefore be regarded as internally consistent and externally supported, but not yet definitive for all clinical settings.

Through WGCNA, differential-expression analysis, and machine-learning feature selection, we reduced a broad PCD-related candidate space to an 8-gene signature. The resulting glmBoost + RF model showed strong performance in the training set and retained an AUC of 0.817 in the independent validation set. We agree that the decline from 0.988 to 0.817 suggests some degree of model optimism and possible overfitting, which is common in high-dimensional biomarker discovery. However, the validation performance remained acceptable, and the model did not undergo performance collapse, supporting its value as a candidate predictive signature that still requires prospective multicenter validation.

SHAP analysis added an interpretable layer to the classifier. PADI4 tended to shift the model toward lower predicted risk, whereas the other model genes tended to increase predicted risk. This pattern suggests that the final signature is not biologically unidirectional but instead captures a balance of potentially protective and risk-associated processes. Because the present analyses are observational, we revised the wording throughout the manuscript to avoid implying causality and to emphasize that the model identifies associations related to the 8-gene signature.

The biological interpretation of the single-cell data also required refinement. ALI is classically associated with alveolar epithelial damage, but endothelial dysfunction is likewise central to vascular leakage, leukocyte trafficking, and loss of barrier integrity. In our scRNA-seq dataset, several model genes were enriched in endothelial cells, and endothelial cells showed extensive crosstalk with natural killer cells, matrix fibroblasts, and pericytes. We therefore interpret endothelial cells as a prominent compartment for PCD-related signaling in this dataset, not as the sole pathogenic cell type or as evidence against epithelial injury. Rather, the data suggest that endothelial PCD programs may represent an important interface between systemic inflammation and lung barrier failure [22–25].

Experimental validation in rodents was intended as supportive evidence rather than direct confirmation of all human molecular events. The bulk cohorts were human, whereas the scRNA-seq dataset and animal validation were rodent, so we harmonized genes by one-to-one ortholog mapping to reduce false correspondence. This conservative approach inevitably discards species-specific immune regulators and may miss biologically relevant non-orthologous signals. Likewise, only 3 of the 8 genes were validated at the protein level. We selected PADI4, POLB, and IFI27 because they ranked highly, were recoverable after homologous mapping, and were technically feasible for follow-up experiments [26–30]. These experiments therefore serve as proof-of-principle support for the signature rather than comprehensive validation of every model gene.

The association between the PCD score and the immune/metabolic microenvironment further suggests that the 8-gene signature captures broader disease-state biology. The high-score group differed in 11 immune-cell signatures and multiple metabolic pathways, indicating coordinated changes rather than isolated marker behavior [31–33]. We accordingly clarified that the PCD score is an ssGSEA-derived score based on the 8 model genes rather than an undefined index.

This study has several limitations. First, the datasets were obtained from public repositories and remain limited in sample diversity. Second, the blood-based human discovery/validation design and the rodent lung scRNA-seq/animal validation design introduce cross-tissue and cross-species heterogeneity. Third, no cross-dataset merging was performed; instead, each dataset was analyzed independently, which minimized inter-cohort batch effects but does not eliminate platform-specific biases within each dataset. Fourth, the PCD analyses rely on curated gene sets and enrichment scores rather than direct functional measurements. Fifth, Western blot normalization used GAPDH as the internal control; because housekeeping-gene stability may change under septic conditions, these protein-level results should be interpreted cautiously and together with the histologic evidence.

In summary, by integrating bulk transcriptomics, single-cell profiling, and in vivo validation, we defined PCD-associated molecular features in sepsis-induced ALI and established an 8-gene classifier with external validation support. The results support a model in which systemic inflammatory stress reshapes PCD-related transcriptional programs, engages both immune and vascular compartments, and contributes to lung injury. These findings provide candidate biomarkers and a framework for future mechanistic and translational studies targeting PCD-related pathways.

## Supporting information

S1 Fig Individual ROC curves for selected model genes. (JPG)

S2 Fig GSEA enrichment plots for model genes. (JPG)

S3 Fig Immune checkpoint gene differences between PCD score groups. (JPG)

S4 Fig TF-mRNA-drug network. (JPG)

S5 Fig Molecular docking results. (JPG)

S6 Fig scRNA-seq quality control and clustering diagnostics. (JPG)

S7 Fig Expression of six model genes across cell types. (JPG)

S8 Fig Endothelial subpopulation analyses and pseudotime trajectories. (JPG)
