# Supplementary figures and images for: Integration of single-cell and bulk RNA sequencing reveals programmed cell death-associated transcriptional programs in sepsis-induced acute lung injury
Source: PLoS One. 2026 Jun 3;21(6):e0349288. doi: 10.1371/journal.pone.0349288 (PMC13232957; doi:10.1371/journal.pone.0349288)

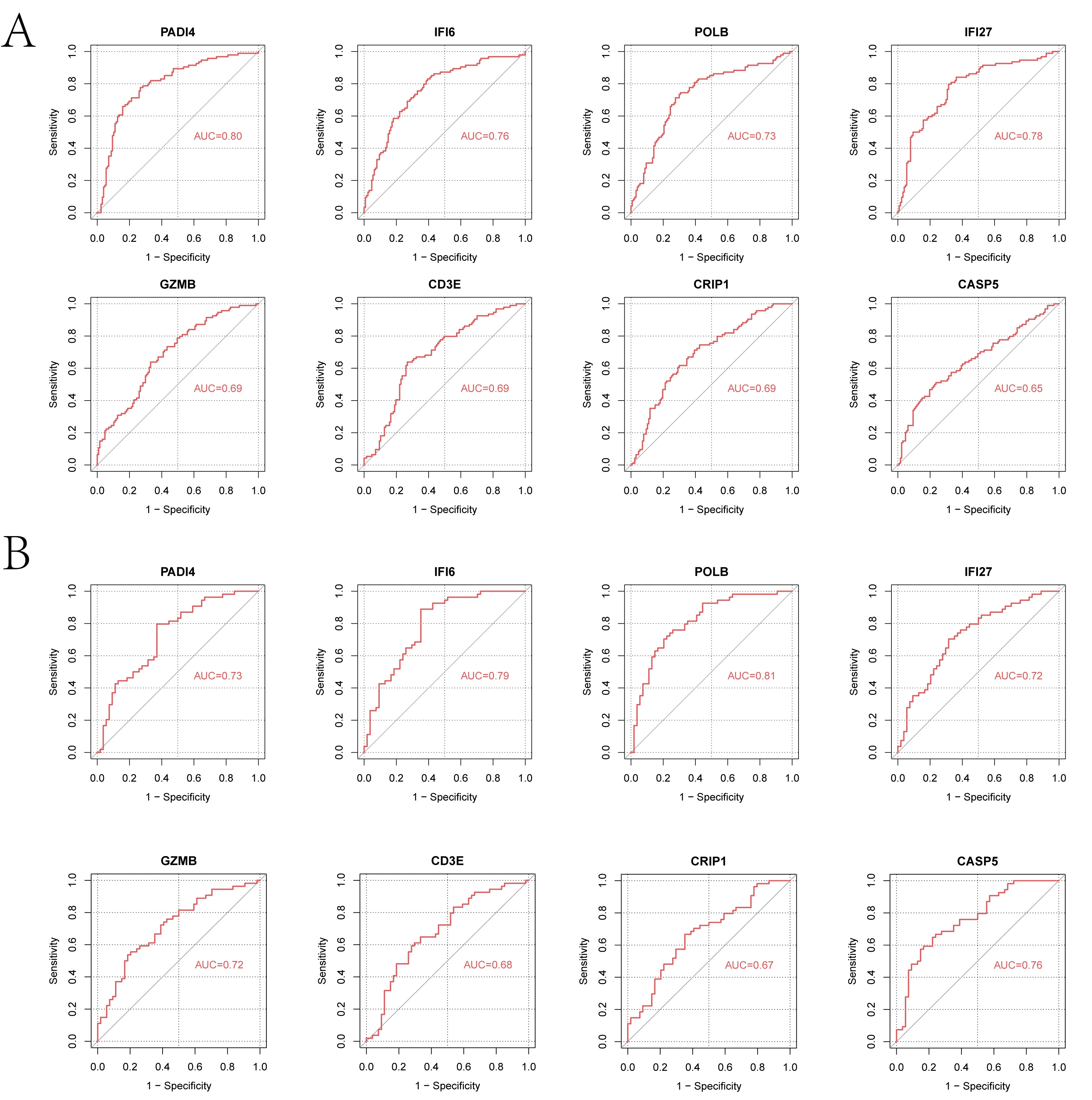

Supplement: S1 Fig — (JPG) [file pone.0349288.s001.jpg]

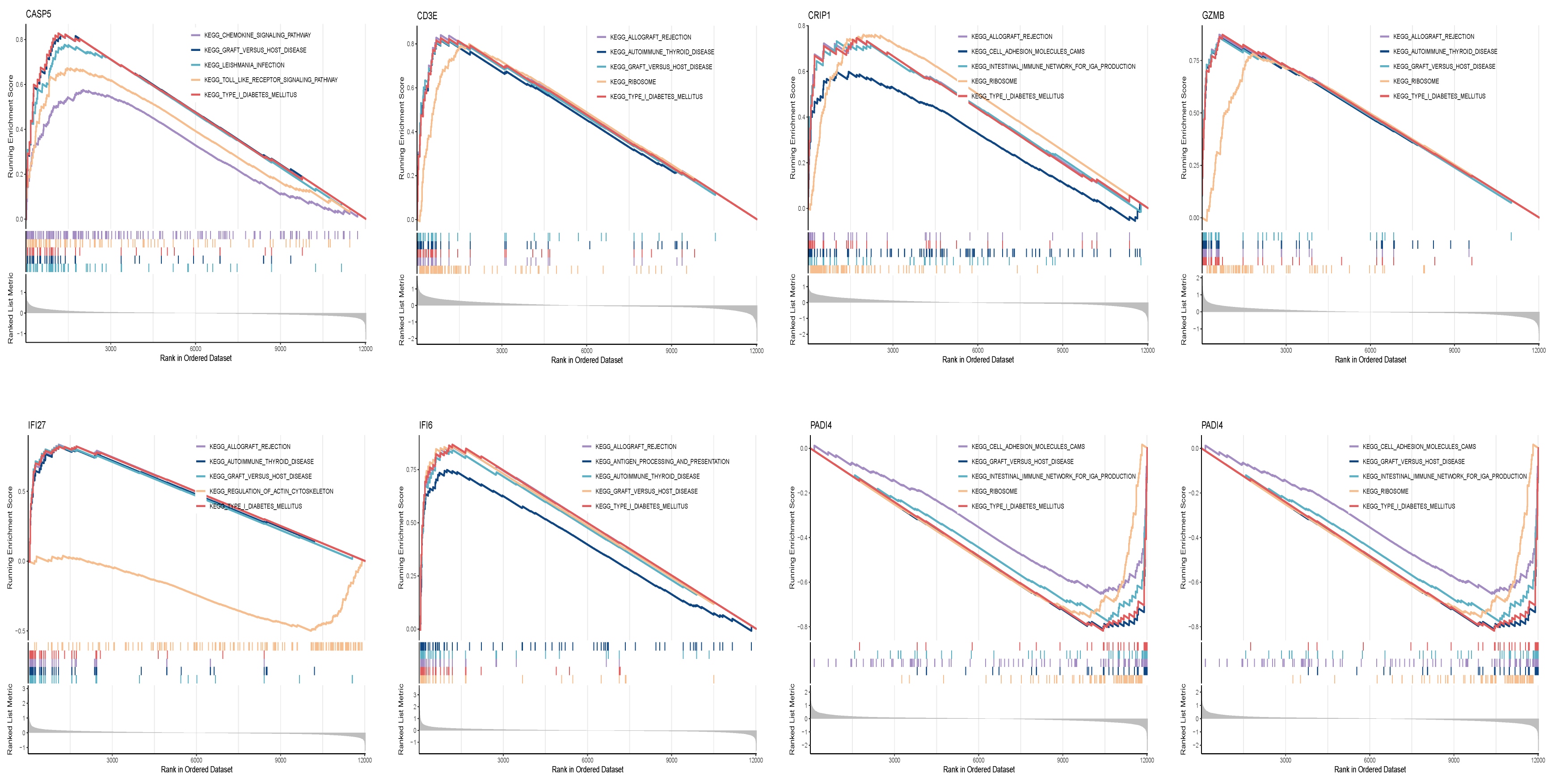

Supplement: S2 Fig — (JPG) [file pone.0349288.s002.jpg]

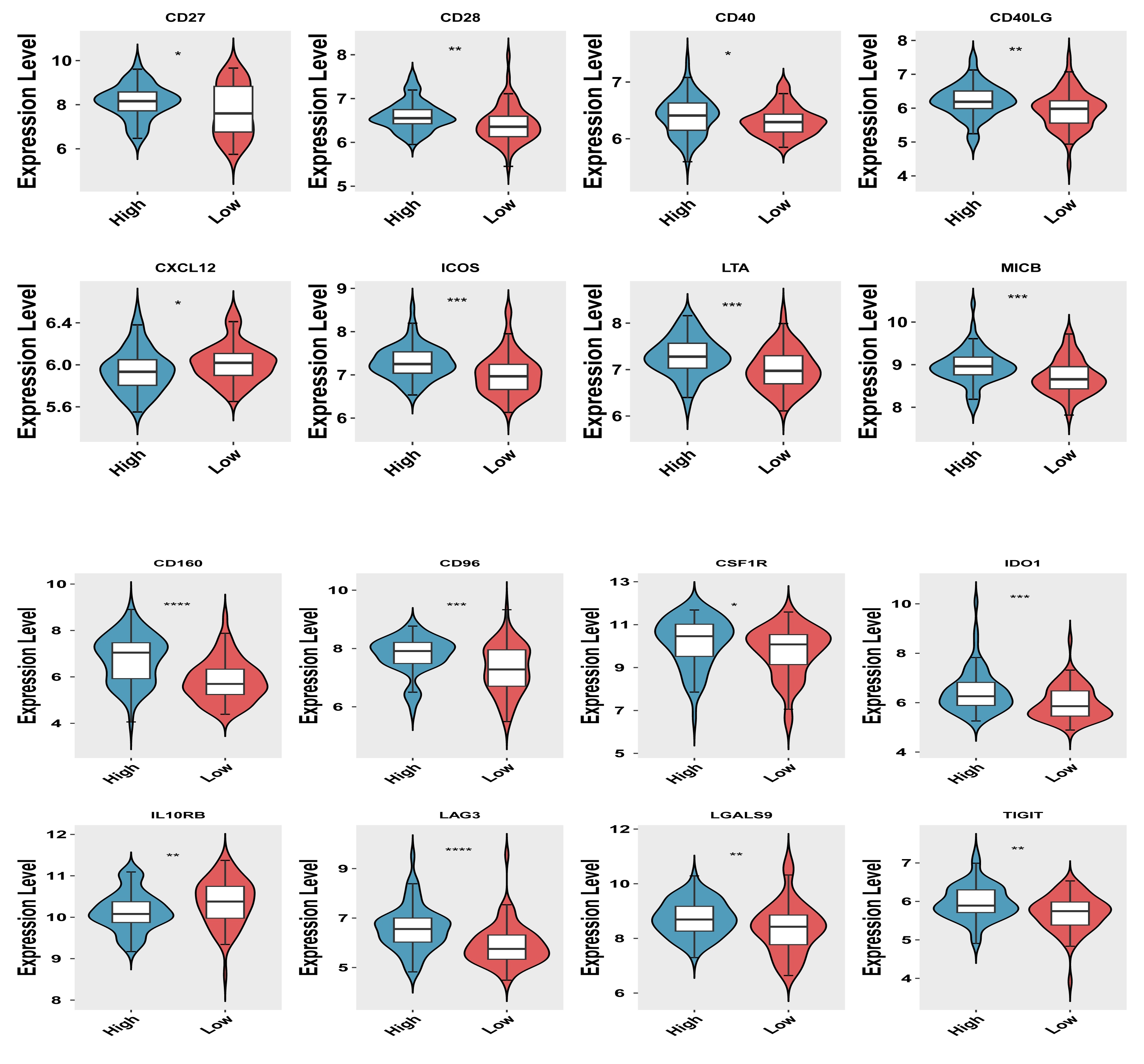

Supplement: S3 Fig — (JPG) [file pone.0349288.s003.jpg]

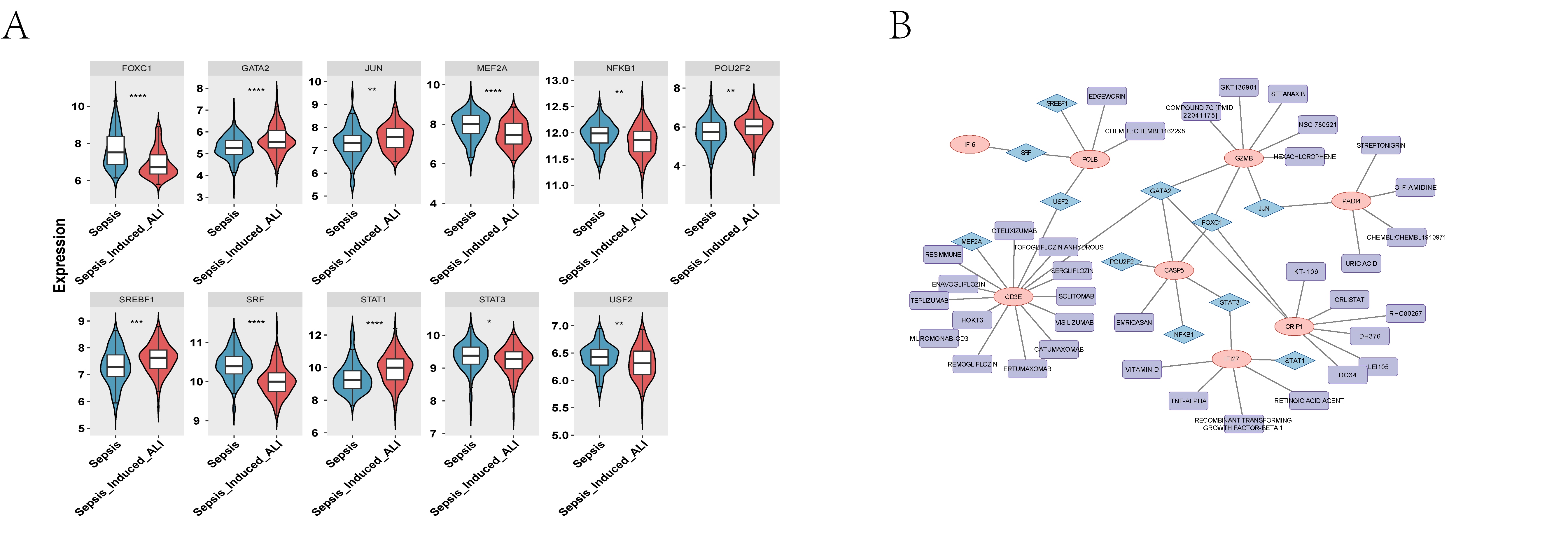

Supplement: S4 Fig — (JPG) [file pone.0349288.s004.jpg]

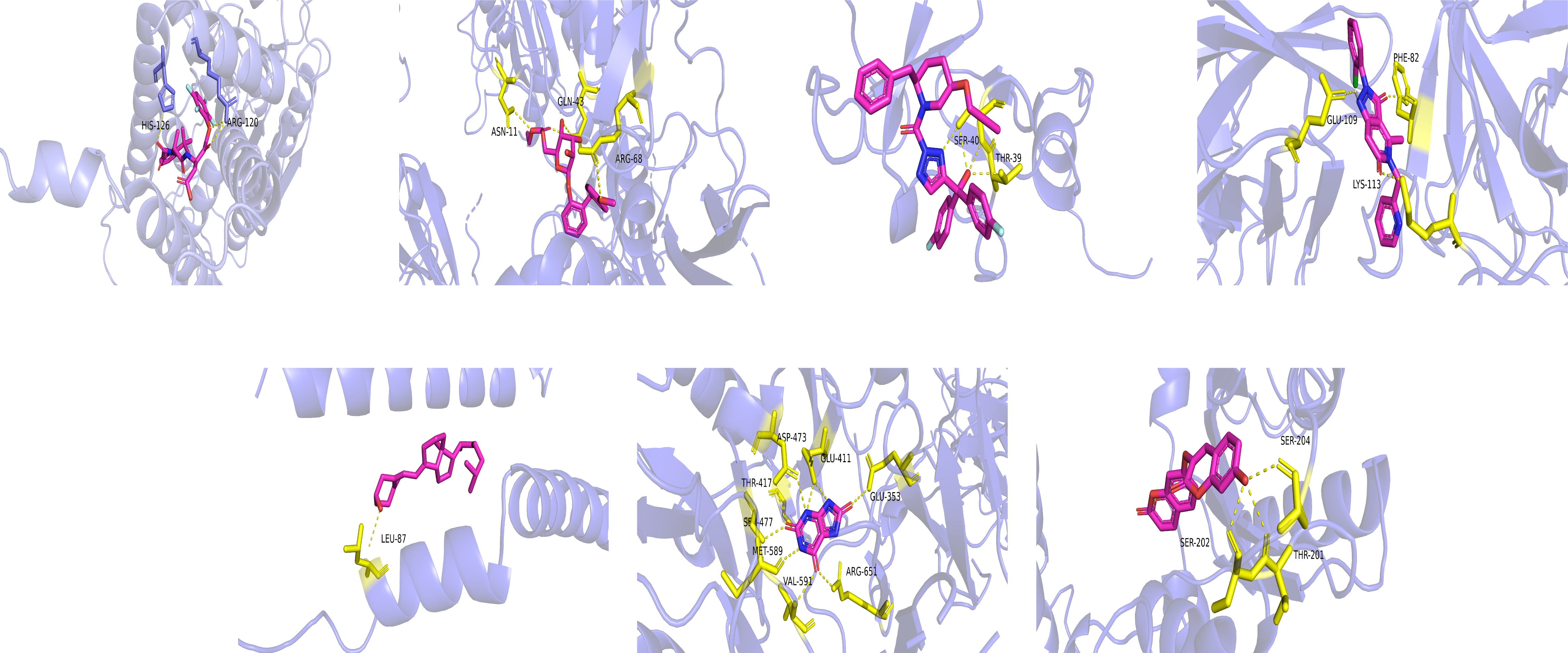

Supplement: S5 Fig — (JPG) [file pone.0349288.s005.jpg]

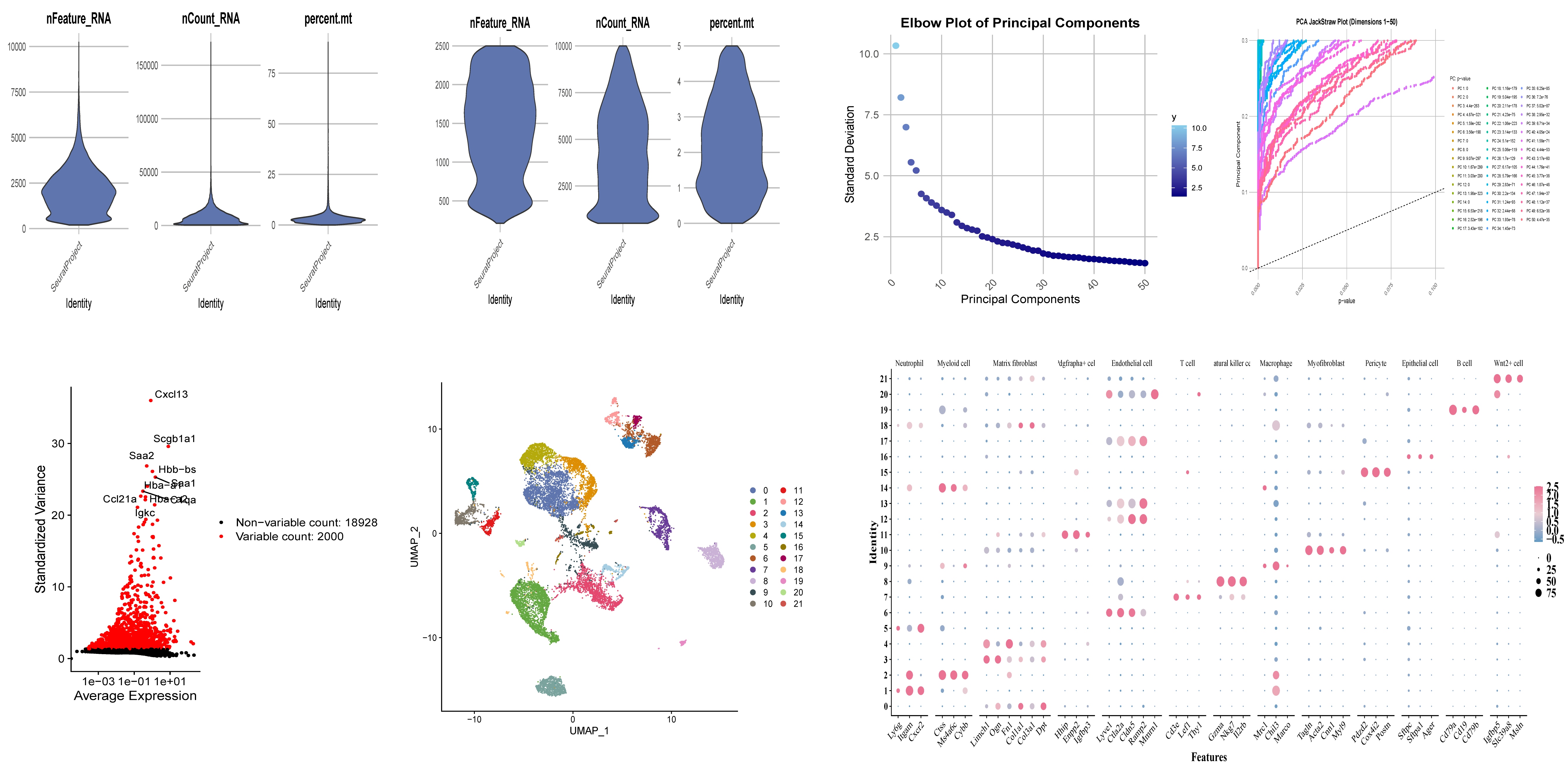

Supplement: S6 Fig — (JPG) [file pone.0349288.s006.jpg]

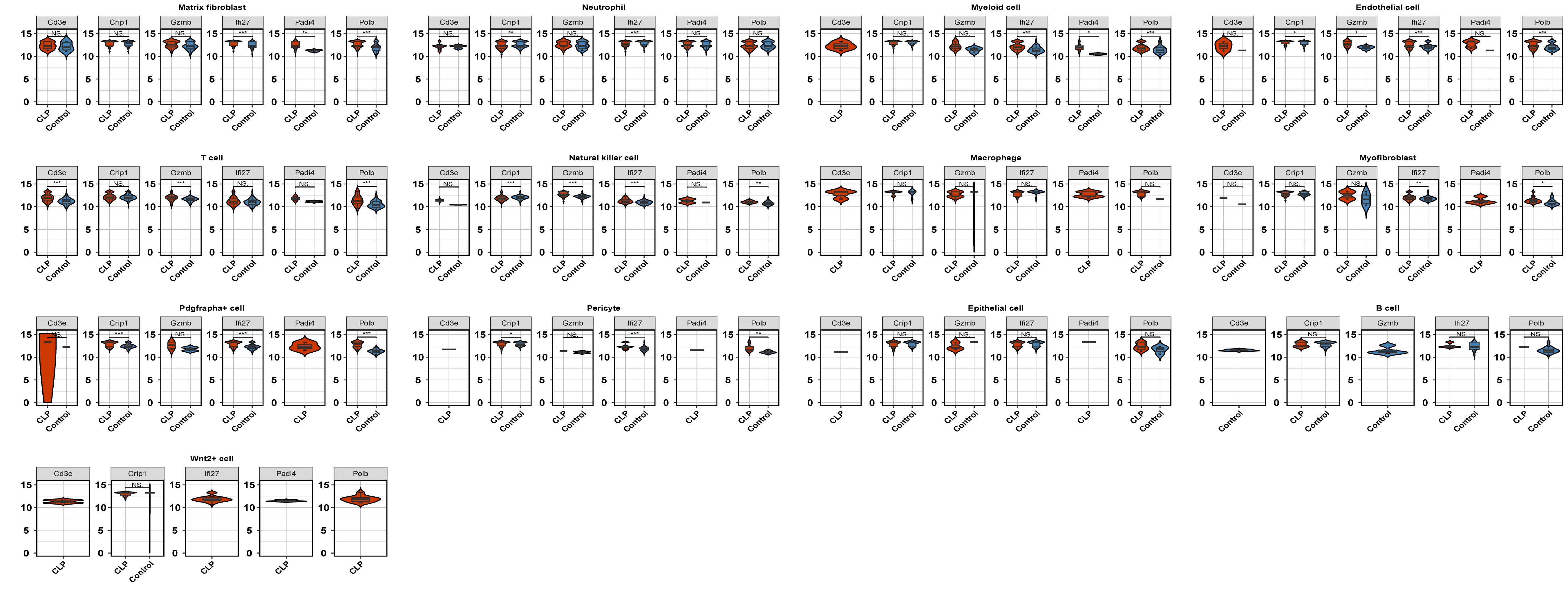

Supplement: S7 Fig — (JPG) [file pone.0349288.s007.jpg]

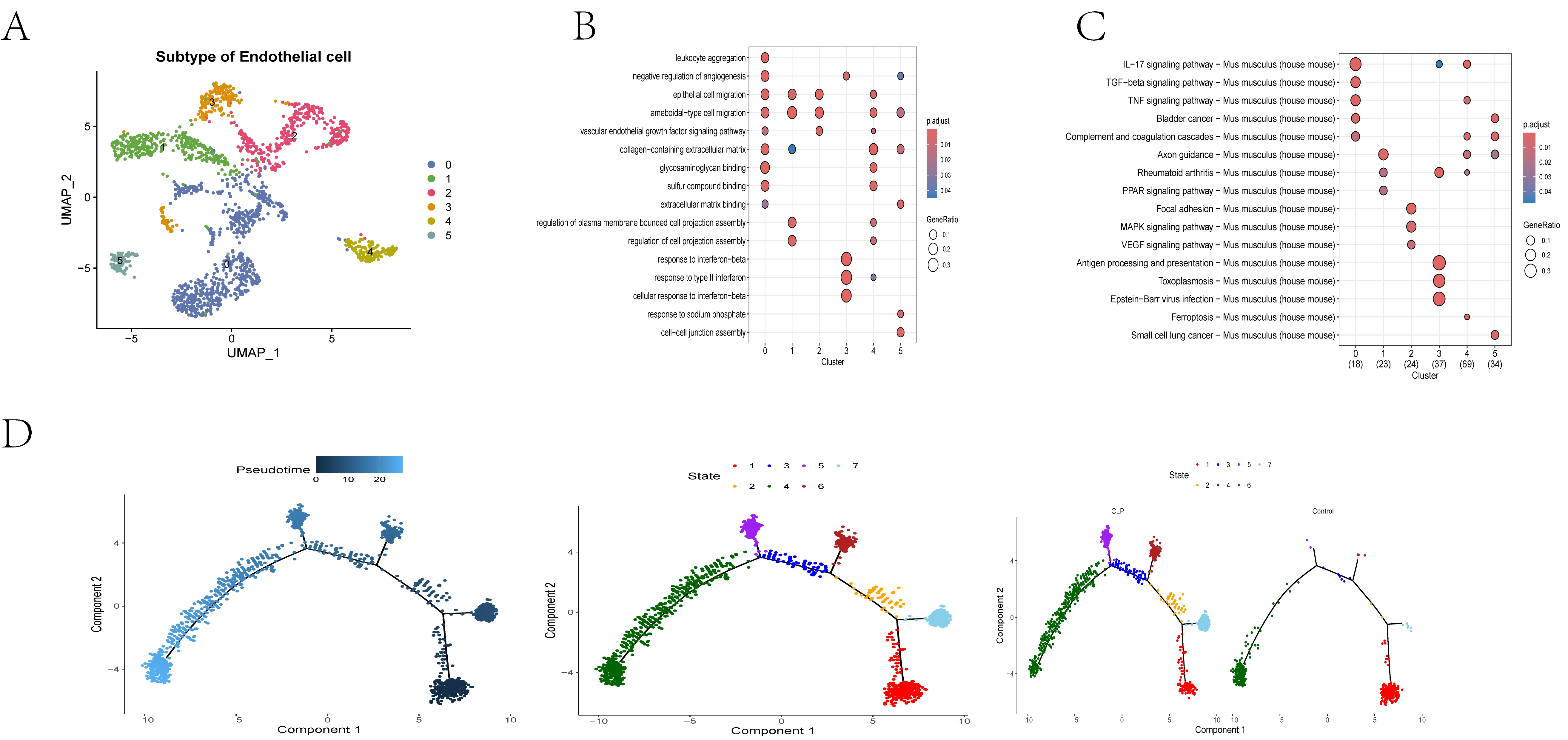

Supplement: S8 Fig — (JPG) [file pone.0349288.s008.jpg]
